# Supplementary material for: Whole genome sequencing of skull-base chordoma reveals genomic alterations associated with recurrence and chordoma-specific survival
Source: Nat Commun. 2021 Feb 3;12:757. doi: 10.1038/s41467-021-21026-5 (PMC7859411; doi:10.1038/s41467-021-21026-5)

Supplementary Material

Supplementary Table 1**:** Known cancer predisposition genes and potential chordoma genes included for germline pathogenic variant evaluation.

ABCB11	*CYLD*	*GPC3*	*PMS2*	*SERPINA1*	*VHL*	*FLNA*	*PLXNB2*
ALK	*DDB2*	*HFE*	*POLD1*	*SETD2*	*WAS*	*GATA3*	*POLE*
APC	*DICER1*	*HMBS*	*POLE*	*SH2D1A*	*WRN*	*GNAS*	*POLRMT*
ARHGAP26	*DIS3L2*	*HRAS*	*POLH*	*SLC25A13*	*WT1*	*HGF*	*PTEN*
ARID1A	*DKC1*	*ITK*	*PRKAR1A*	*SMAD4*	*XPA*	*HIST1H1E*	*PTPRC*
ATM	*DOCK8*	*KIT*	*PRSS1*	*SMARCA4*	*XPC*	*HLA-A*	*RPL22*
AXIN2	*EGFR*	*LYST*	*PTCH1*	*SMARCB1*	*STAB1*	*IDH1*	*RUNX1*
BAP1	*ELANE*	*MAX*	*PTEN*	*SMARCE1*	*AC007461.1*	*INPPL1*	*SETD2*
BLM	*ERBB4*	*MEN1*	*PTPN11*	*SOS1*	*UBBP4*	*IRF2*	*SF3B1*
BMPR1A	*ERCC2*	*MET*	*PTPRD*	*SRY*	*EIF5AL1*	*JAK1*	*SPTA1*
BRCA1	*ERCC3*	*MLH1*	*RAD51C*	*STAT3*	*APOB*	*KDM5C*	*STK11*
BRCA2	*ERCC4*	*MSH2*	*RAD51D*	*STK11*	*ARID2*	*KMT2A*	*TP53*
BRIP1	*ERCC5*	*MSH6*	*RB1*	*SUFU*	*B2M*	*LATS2*	*TXNIP*
BUB1B	*EXT1*	*MTAP*	*RECQL4*	*T*	*BCOR*	*MAP3K4*	*USP9X*
CBL	*EXT2*	*MUTYH*	*RET*	*TERT*	*CACNA1A*	*MAX*	*VHL*
CDC27	*FAH*	*NBN*	*RHBDF2*	*TGFBR1*	*CDKN2A*	*MED12*	*ZFHX3*
CDC73	*FANCA*	*NF1*	*RMRP*	*TMEM127*	*CDKN2C*	*MGA*	*ZNF750*
CDH1	*FANCC*	*NF2*	*RUNX1*	*TNFRSF6*	*CSDE1*	*MLH1*	
CDK4	*FANCG*	*PALB2*	*SBDS*	*TP53*	*DAZAP1*	*NIPBL*	
CDKN1B	*FH*	*PBRM1*	*SDHA*	*TRIM37*	*EGFR*	*NUP133*	
CDKN2A	*FLCN*	*PDGFRA*	*SDHAF2*	*TSC1*	*EP300*	*PBRM1*	
CEBPA	*GATA2*	*PHOX2B*	*SDHB*	*TSC2*	*EPAS1*	*PDGFRA*	
CHEK2	*GBA*	*PIK3CA*	*SDHC*	*UROD*	*ERBB4*	*PGR*	
COL7A1	*GJB2*	*PIK3R1*	*SDHD*	*USP9X*	*FAT1*	*PLCG1*	

Supplementary Figure 1: Average sequencing depth for normal and tumor (80 primary + 11 recurrent) samples among 80 chordoma patients

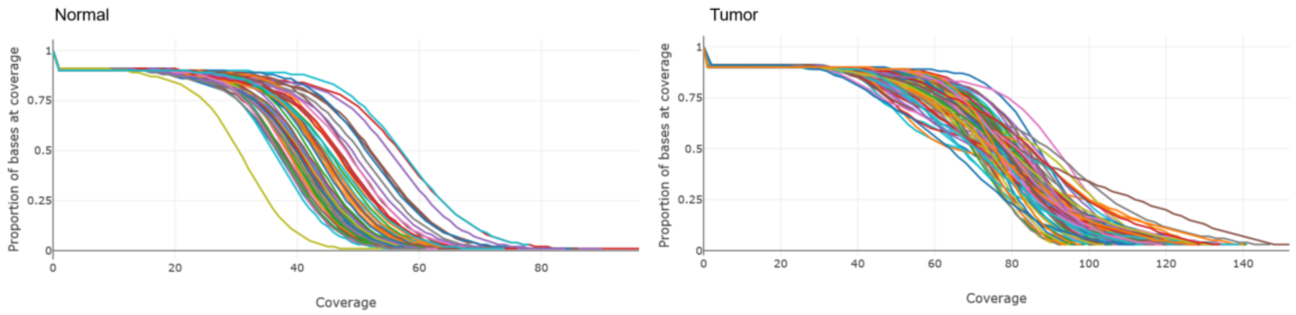

Supplementary Table 2A: The distribution of single-base substitutions (SBS) observed in 80 primary tumors

SBS signature	Mean	Range	
SBS1	0.10	0.04	0.20
SBS2	0.01	0	0.15
SBS5	0.68	0	0.95
SBS8	0.10	0	0.32
SBS13	0.01	0	0.17
SBS40	0.05	0	0.70
SBS44	0.01	0	0.29

Supplementary Table 2B: The distribution of *de novo* indel (ID) signatures observed in 80 primary tumors

ID signature	Mean	Range	
A	0.27	0	1
B	0.20	0	0.74
C	0.08	0	1
D	0.11	0	1
E	0.34	0	0.88

Supplementary Figure 2: Germline *TBXT* duplication in one chordoma patient. *TBXT* gene location: chr6:166571146-166582157 (hg19). Red indicates the duplication region.

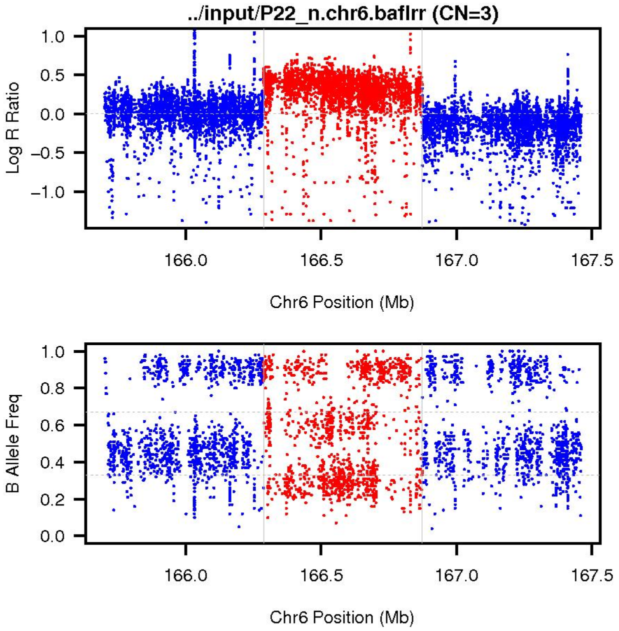

Supplementary Table 3: Comparison of mutational profiles of chordoma patients to those of multiple other cancer types using MutaGene. Somatic mutations for each chordoma tumor sample were input to the program separately and compared to those of 9,450 cancer samples included in MutaGene. Ranking indicates the level of similarity between the input chordoma sample and each MutaGene cancer type. Numbers in the table reflect the number of chordoma samples (out of 80) in each ranking category for each cancer type.

Cancer type	1	2	3	4	5	6	7	8	9	10
Bladder Urothelial Carcinoma					1		1			
Bone Cancer	2	6	5	11	25	20	5	4	1	1
Brain Glioblastoma Multiforme							1			2
Brain Lower Grade Glioma		1	1		1				5	42
Breast Cancer	2						1		1	1
Chronic Lymphocytic Leukemia	21	11	11	18	6	7	1	1	1	1
Colon Adenocarcinoma								1	1	
Esophageal Adenocarcinoma								1		
Esophageal Squamous Carcinoma		2	1				1	1	3	11
Gastric Cancer	2									
GCB Lymphomas				1		2	4	11	52	4
Hepatocellular Carcinoma			2	3	7	11	15	24	5	1
Kidney Renal Clear Cell Carcinoma	14	26	18	10	4	5		1		2
Kidney Renal Papillary Cell Carcinoma	38	24	13	3						
Lung Squamous Cell Carcinoma							1	2	1	2
Ovarian Serous Cystadenocarcinoma		1	4	11	17	25	15	1	3	1
Pancreatic Cancer					1		1	2	2	7
Pancreatic Cancer Endocrine neoplasms										3
Renal Cancer	1	8	24	20	15	7			1	
Thyroid Cancer				1	3	3	34	31	4	1
Triple Negative Breast Cancer		1	1							1
Uterine Corpus Endometrial Carcinoma				2						

Supplementary Figure 3: Tumor mutational burden (TMB) in chordoma tumors in relation to other tumor types included in The Cancer Genome Atlas (TCGA)

Tumor types were ordered by median TMB values (Log10), from high to low. SKCM: skin cutaneous melanoma; LUSC: lung squamous cell carcinoma; LUAD: lung adenocarcinoma; BLCA: bladder urothelial carcinoma; STAD: stomach adenocarcinoma; COAD: colon adenocarcinoma; HNSC: head and neck squamous cell carcinoma; READ: rectum adenocarcinoma; DLBC: lymphoid neoplasm diffuse large B-cell adenocarcinoma; ESCA: esophageal carcinoma; OV: ovarian serous cystadenocarcinoma; LIHC: liver hepatocellular carcinoma; CESC: cervical squamous cell carcinoma and endocervical adenocarcinoma; UCEC: uterine corpus endometrial carcinoma; KIRP: kidney renal papillary cell carcinoma; GBM: glioblastoma multiforme; KIRC: kidney renal clear cell carcinoma; UCS: uterine carcinosarcoma; BRCA: breast invasive carcinoma; PAAD: pancreatic adenocarcinoma; SARC: sarcoma; LGG: brain lower grade glioma; PRAD: prostate adenocarcinoma; CHOL: cholangiocarcinoma; MESO: mesothelioma; ACC: adrenocortical carcinoma; Chordoma; KICH: kidney chromophobe; UVM: uveal melanoma; TGCT: testicular germ cell tumors; THYM: thymoma; PCPG: pheochromocytoma and paraganglioma; THCA: thyroid carcinoma; LAML: acute myeloid leukemia. The number of samples sequenced for each study is indicated on the top of the figure.

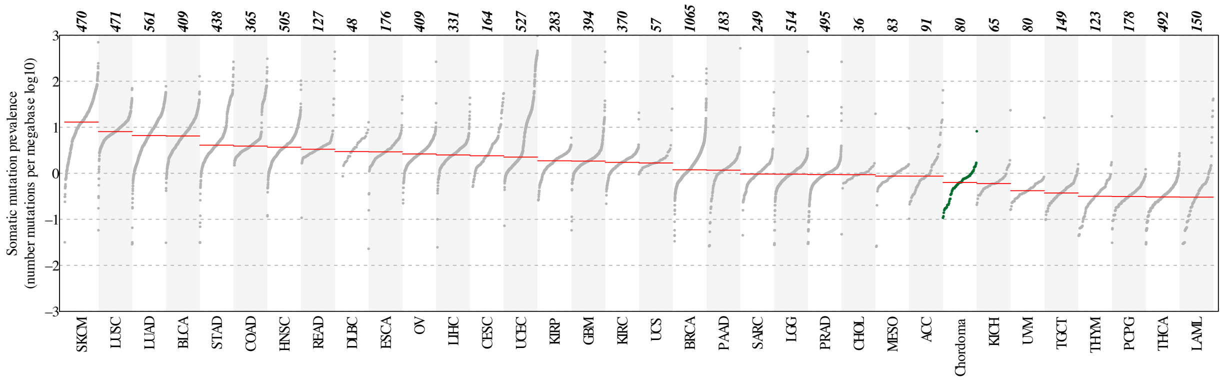

Supplementary Table 4: Percentage of genome showing MCN (major allele copy number) greater than or equal to two.

sample.id	%genome with MCN≥2	sample.id	%genome with MCN≥2	sample.id	%genome with MCN≥2	sample.id	%genome with MCN≥2
primary_P01	0.21	primary_P21	0.04	primary_P41	0	primary_P61	0.24
primary_P02	0.24	primary_P22	0.22	primary_P42	0.04	primary_P62	0.26
primary_P03	0.11	primary_P23	0	primary_P43	0.94	primary_P63	0.25
primary_P04	0.01	primary_P24	0.04	primary_P44	0.2	primary_P64	0.18
primary_P05	0.09	primary_P25	0.13	primary_P45	0.09	primary_P65	0
primary_P06	0.23	primary_P26	0	primary_P46	0	primary_P66	0.39
primary_P07	0	primary_P27	0	primary_P47	0	primary_P67	0.15
primary_P08	0.13	primary_P28	0	primary_P48	0	primary_P68	0
primary_P09	0.04	primary_P29	0	primary_P49	0	primary_P69	0
primary_P10	0.01	primary_P30	0	primary_P50	0.07	primary_P70	0
primary_P11	0.07	primary_P31	0.95	primary_P51	0.95	primary_P71	0.25
primary_P12	0.03	primary_P32	0.02	primary_P52	0.07	primary_P72	0
primary_P13	0	primary_P33	0.01	primary_P53	0.17	primary_P73	0
primary_P14	0.05	primary_P34	0.12	primary_P54	0.02	primary_P74	0.01
primary_P15	0.07	primary_P35	0	primary_P55	0	primary_P75	0
primary_P16	0.12	primary_P36	0	primary_P56	0.02	primary_P76	0.11
primary_P17	0	primary_P37	0.13	primary_P57	0.95	primary_P77	0.11
primary_P18	0.02	primary_P38	0.89	primary_P58	0	primary_P78	0.61
primary_P19	0.18	primary_P39	0.86	primary_P59	0.05	primary_P79	0.24
primary_P20	0	primary_P40	0	primary_P60	0.12	primary_P80	0.01

Supplementary Figure 4: Mutational signatures identified in 80 primary tumors. Bar graphs show the distribution of de novo single-base substitution (SBS) (panel A) and indel (panel B) signatures. Tables below the bar graphs show cosine similarities between de novo identified signatures and COSMIC signatures.

A: Nine *de novo* single-base substitution (SBS) mutational patterns

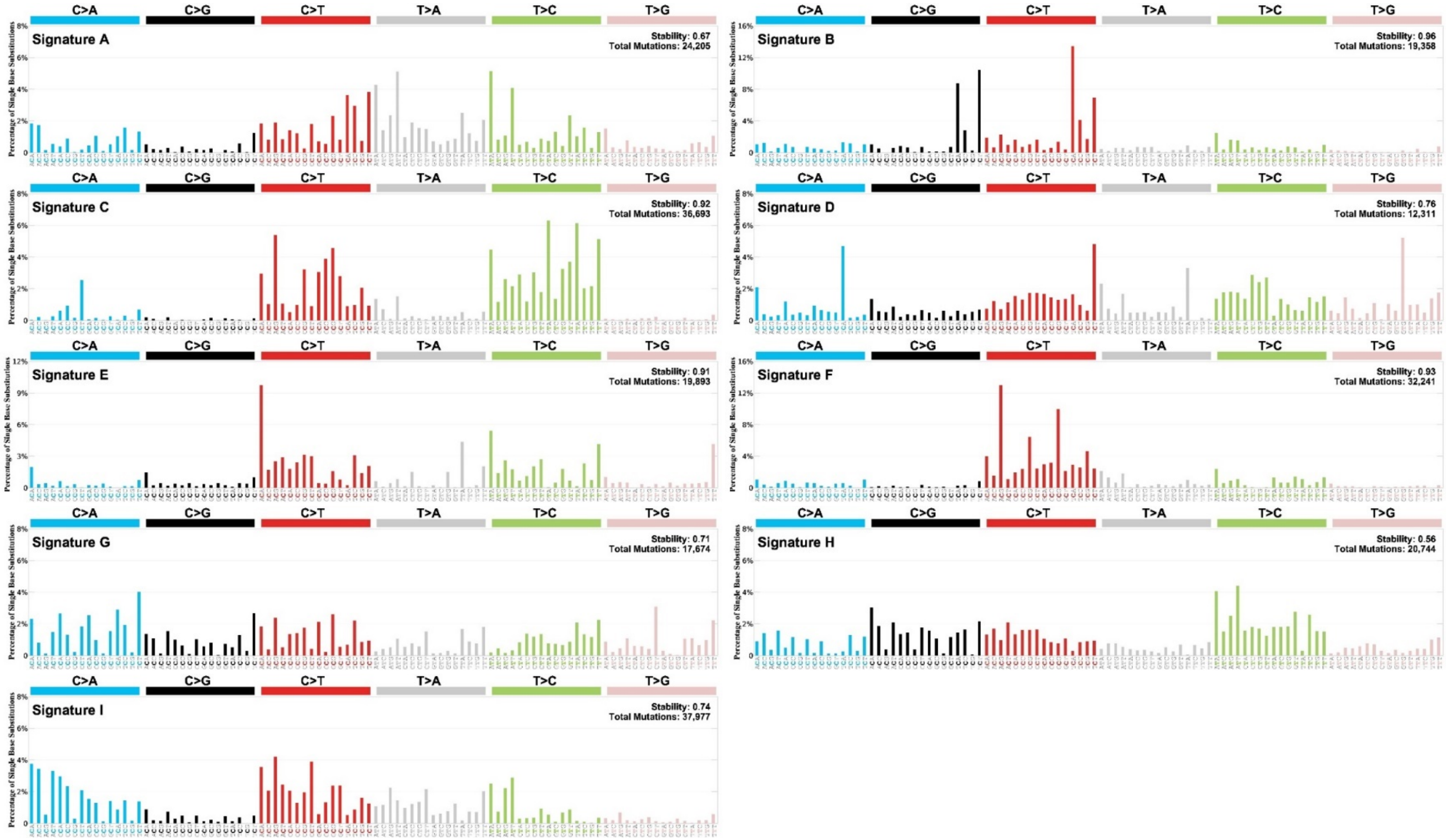

De novo extracted	COSMIC SBS Signatures[a]	Similarity[b]
Signature 96-A	Signature SBS1 (1.96%) & Signature SBS5 (83.56%) & Signature SBS34 (14.48%)	0.86
Signature 96-B	Signature SBS1 (4.14%) & Signature SBS2 (23.16%) & Signature SBS5 (45.54%) & Signature SBS13 (27.16%)	0.99
Signature 96-C	Signature SBS1 (12.96%) & Signature SBS5 (11.86%) & Signature SBS12 (31.98%) & Signature SBS21 (13.40%) & Signature SBS44 (29.80%)	0.98
Signature 96-D	Signature SBS1 (1.34%) & Signature SBS5 (57.76%) & Signature SBS43 (17.04%) & Signature SBS47 (16.24%) & Signature SBS52 (7.62%)	0.92
Signature 96-E	Signature SBS1 (4.72%) & Signature SBS5 (75.82%) & Signature SBS57 (19.46%)	0.84
Signature 96-F	Signature SBS1 (35.12%) & Signature SBS5 (64.88%)	0.96
Signature 96-G	Signature SBS1 (6.98%) & Signature SBS9 (20.86%) & Signature SBS18 (18.32%) & Signature SBS40 (53.84%)	0.93
Signature 96-H	Signature SBS1 (0.36%) & Signature SBS5 (65.76%) & Signature SBS39 (33.88%)	0.92
Signature 96-I	Signature SBS1 (8.62%) & Signature SBS5 (34.90%) & Signature SBS8 (48.30%) & Signature SBS19 (8.18%)	0.96

[a]: The best linear combination of existing COSMIC signatures that approximates the detected *de novo* SBS in our data. [b]: The cosine similarity between our detected *de novo* mutational signatures and the linear combination of existing COSMIC mutation signatures.

B: Five major *de novo* indel signatures (ID)

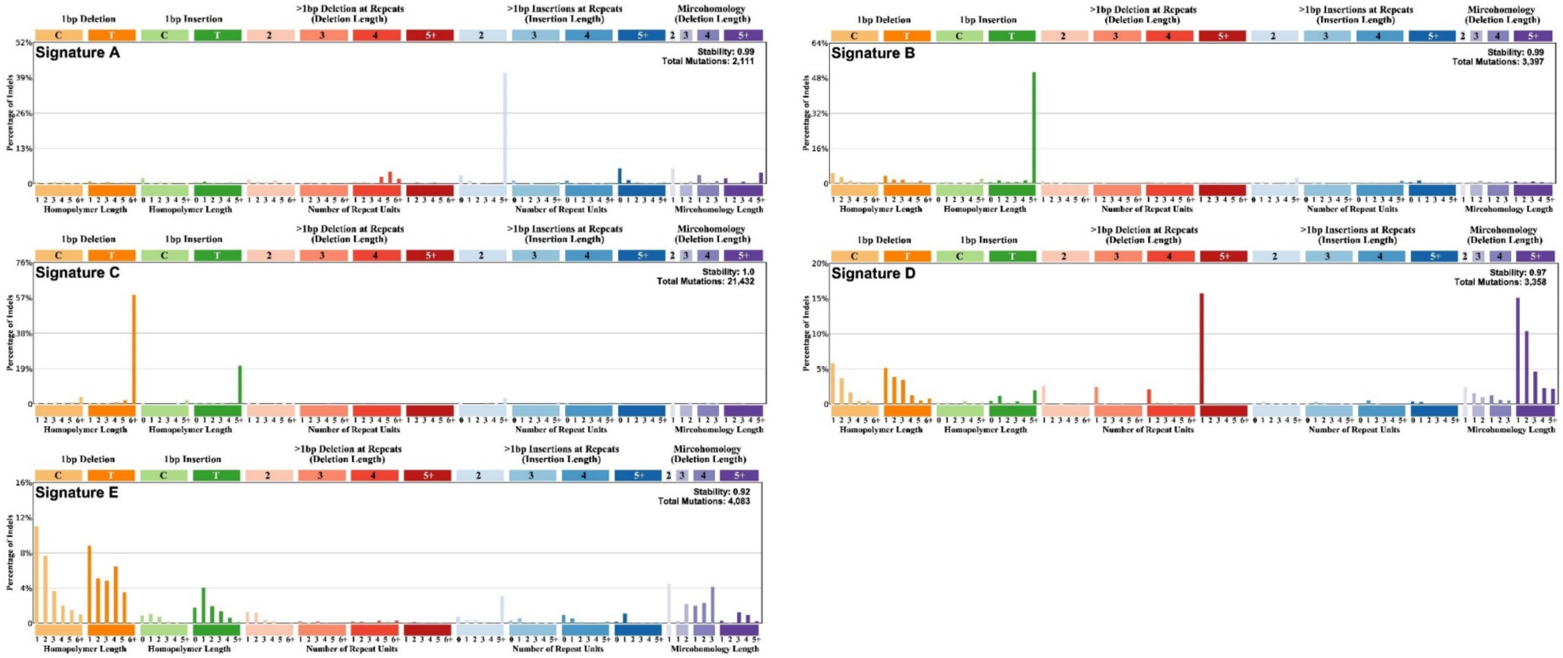

De novo extracted	COSMIC Indel Signatures[a]	Similarity[b]
Signature INDEL-A		
Signature INDEL-B	Signature ID1 (66.06%) & Signature ID3 (15.20%) & Signature ID5 (18.74%)	0.99
Signature INDEL-C	Signature ID1 (24.02%) & Signature ID2 (75.98%)	1
Signature INDEL-D	Signature ID5 (28.04%) & Signature ID6 (10.80%) & Signature ID8 (61.16%)	0.98
Signature INDEL-E	Signature ID3 (23.00%) & Signature ID4 (9.42%) & Signature ID5 (40.02%) & Signature ID9 (27.56%)	0.93

[a]: The best linear combination of existing COSMIC signatures that approximates the detected *de novo* SBS in our data. [b]: The cosine similarity between our detected *de novo* mutational signatures and the linear combination of existing COSMIC mutation signatures.

Supplementary Table 5: High-confidence chromothripsis events identified by ShatterSeek

ID	Position	#SVs in sample	#CN segments	Pval chr. breakp. enrich.	Pval exponential dist. breakpoints	Pval fragment joins	Links with other chrs
P09	4:16473677−186968109	96	16	0	0	0.01	11:54996720−131215637;3:33853751−82347706; chrs 7:80822537−114954467;8:117486242−137195855
P09	7:80822537−114954467	96	9	0	0	0.02	11:54996720−131215637;4:16473677−186968109
P09	22:18983600−50619038	96	6	NA	0	0.01	
P16	3:1236917−169015428	572	24	0	0	0	10:351295−111311574;
P16	6:56533939−64320531	572	6	0.01	0	0.03	
P16	10:351295−111311574	572	18	0	0	0	
P25	3:4902901−148147061	29	12	0	0	0.01	
P63	6:90080348−170842513	109	76	NA	0	0	
P66	11:15626344−38061210	44	5	0	0	0.01	10:43740177−46096635
P79	1:1481021−246175291	170	23	0	0	0	20:14410348−62070724
P79	19:35069904−54840360	170	17	0.01	0	0	
P80	5:13050718−40240915	34	6	0.06	0	0	1:1481021−246175291;13:19749310−52735863

Supplementary Figure 5: Comparison of clonal and subclonal mutation signatures. (A): Scatter plots of contribution of signatures of clonal SNVs vs. subclonal SNVs for 10 signatures. Each point represents one tumor. Pearson correlation coefficients are shown on the figure for each signature. (B): Average contribution of mutation signatures based on clonal and subclonal mutations. P values were obtained from Wilcoxon test (two-sided) for the difference of contribution of clonal and subclonal signatures. Figures were plotted based on n=80 skull-base chordoma samples. Differences were not statistically significant after the correction for multiple testing for any of the examined signatures.

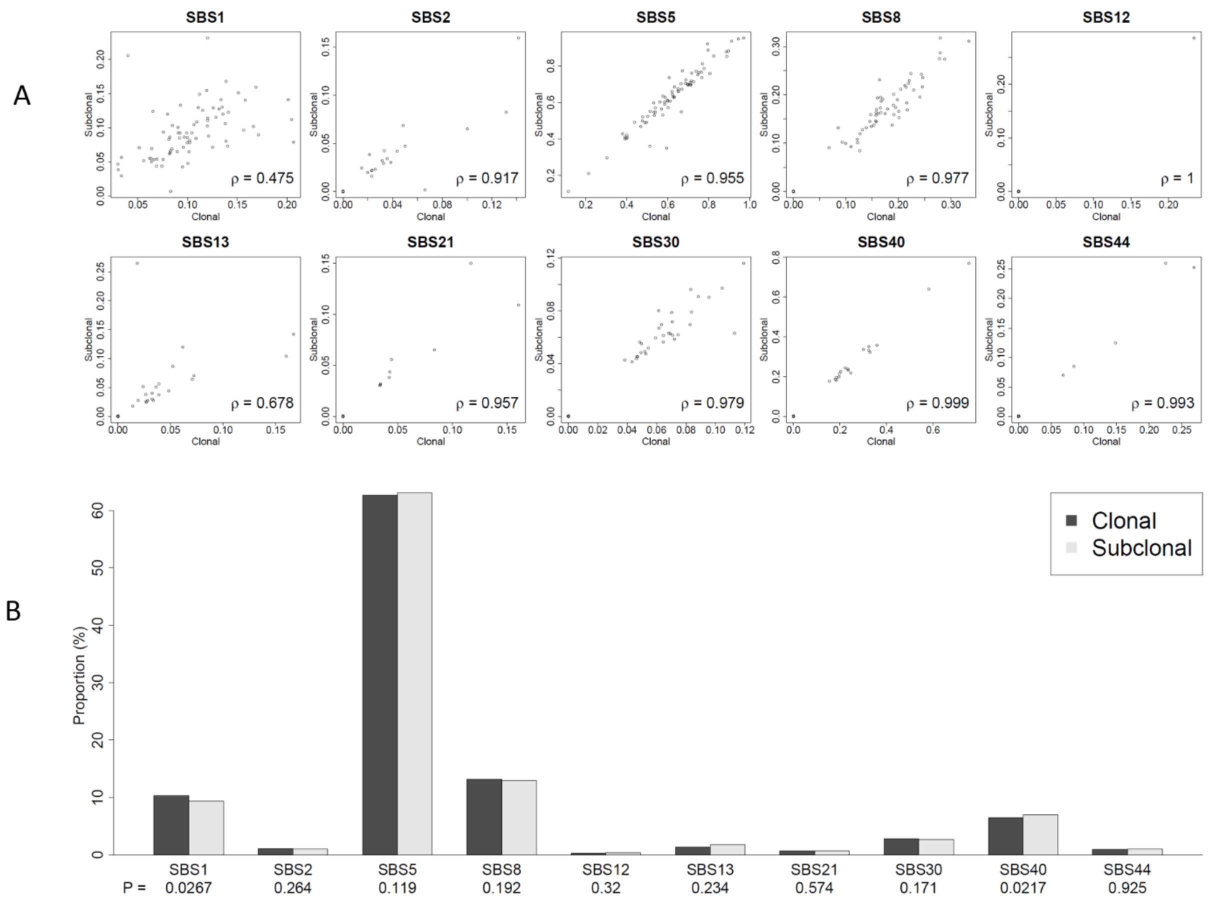

Supplementary Table 6: Associations between genomic features and chordoma specific survival (CSS) and recurrence free survival (RFS), based on Cox's proportional hazard model. Models were adjusted for age, sex, pre- and post-surgery radiation therapy. HR=hazard ratio; Lower and Upper=95% confidence interval; SCNA: somatic copy number alteration, reference group is SCNA group 4. *Comparing patients harboring any of these events to those harboring none of these events.

A. CSS.

Genomic features	HR	Lower	Upper	P
PBRM1+	4.79	1.57	14.59	0.0058
CDKN2A/B+	0.88	0.20	3.92	0.86
SCNA_Group1[a]	2.21	0.39	12.43	0.37
SCNA_Group2[a]	3.39	0.62	18.48	0.16
SCNA_Group3[a]	1.90	0.25	14.33	0.53
SCNA_Group5[a]	1.22	0.11	14.04	0.87
1p deletion	0.94	0.31	2.83	0.92
3p deletion	3.94	1.09	14.29	0.04
3q deletion	2.81	0.89	8.89	0.08
4p deletion	1.06	0.33	3.38	0.93
4q deletion	1.04	0.31	3.42	0.95
9p deletion	2.01	0.56	7.17	0.28
9q deletion	2.46	0.80	7.55	0.12
10p deletion	2.25	0.75	6.80	0.15
10q deletion	2.28	0.75	6.92	0.15
13q deletion	2.79	0.85	9.23	0.09
14q deletion	3.21	0.93	11.06	0.06
18p deletion	3.03	1.08	8.55	0.04
18q deletion	3.08	1.09	8.75	0.03
22q deletion	5.88	1.85	18.68	0.0027
1q amplification	1.51	0.45	5.09	0.50
7p amplification	1.47	0.54	4.03	0.45
7q amplification	1.87	0.63	5.61	0.26
9p21.3	1.44	0.42	4.88	0.56
9p11.2	1.33	0.42	4.16	0.62
9q21.11	1.80	0.61	5.32	0.29
PBRM1+ or 22q deletion*	10.55	2.81	39.64	0.001

B. RFS.

Genomic features	HR	Lower	Upper	P
PBRM1+	5.72	2.68	12.19	$6.4x10^{-6}$
CDKN2A/B+	1.68	0.66	4.31	0.28
SCNA_Group1[a]	2.55	1.06	6.11	0.04
SCNA_Group2[a]	1.77	0.78	3.99	0.17
SCNA_Group3[a]	2.22	0.93	5.30	0.07
SCNA_Group5[a]	1.39	0.48	4.04	0.54
1p deletion	1.41	0.77	2.57	0.26
3p deletion	1.53	0.86	2.73	0.15
3q deletion	1.84	1.05	3.24	0.03
4p deletion	1.66	0.83	3.33	0.15
4q deletion	1.84	0.89	3.81	0.10
9p deletion	3.36	1.74	6.94	0.0003
9q deletion	3.99	2.08	7.65	$3.06x10^{-05}$
10p deletion	1.71	0.97	3.04	0.07
10q deletion	1.56	0.89	2.71	0.12
13q deletion	1.08	0.62	1.88	0.78
14q deletion	2.13	1.20	3.79	0.01
18p deletion	2.39	1.36	4.20	0.002
18q deletion	2.65	1.51	4.65	0.001
22q deletion	3.74	1.89	7.38	0.0001
1q amplification	1.53	0.81	2.91	0.19
7p amplification	1.68	0.97	2.93	0.07
7q amplification	1.49	0.85	2.59	0.16
9p21.3	2.65	1.42	4.93	0.002
9p11.2	2.54	1.38	4.67	0.003
9q21.11	3.63	1.97	6.69	$3.4x10^{-5}$
PBRM1+ or 9q21.11 deletion or 22q deletion*	4.22	2.34	7.62	$1.77x10^{-6}$

C. Sensitivity analyses

CSS	PBRM1		9p21.3		9q		22q	
	HR	p value	HR	p value	HR	p value	HR	p value
Final model[a]	4.79	0.01	1.44	0.56	1.80	0.29	5.88	0.003
Final model + TMB[b]	5.13	0.01	1.43	0.59	1.85	0.29	6.16	0.003
Final model + SCNA[c]	6.02	0.01	1.11	0.89	2.15	0.28	6.31	0.01
Final model + Ki67	46.05	0.003	10.71	0.02	10.70	0.02	5.04	0.04
Final model + GRR[d]	3.70	0.02	1.26	0.69	1.60	0.38	4.51	0.01
Final model[e]	21.88	0.001	1.40	0.71	1.42	0.69	15.93	0.01

RFS	PBRM1		9p21.3		9q		22q	
	HR	p value	HR	p value	HR	p value	HR	p value
Final model[a]	5.72	6.44E-06	2.65	0.002	3.63	3.44E-05	3.74	0.0001
Final model + TMB[b]	5.69	7.07E-06	2.61	0.003	3.62	5.43E-05	3.72	2.00E-04
Final model + SCNA[c]	5.17	4.58E-05	2.27	0.03	4.25	4.16E-04	3.26	0.003
Final model + Ki67	6.79	0.001	3.24	0.01	4.36	0.002	3.16	0.02
Final model + GRR[d]	5.13	3.40E-05	2.55	0.003	3.54	3.70E-05	3.54	3.00E-04
Final model[e]	7.21	0.004	2.96	0.004	3.01	0.04	5.53	2.00E-04

[a]- Covariates: age, gender, pre-surgery radiation therapy (yes vs. no), post-surgery radiation therapy (yes vs. no)

[b]- Tumor mutation burden

[c]- Somatic copy number alterations

[d]- Gross resection rate

[e]- Restricting to patients who were diagnosed and operated in our hospital and did not have any treatment prior to surgeries

Supplementary Figure 6**:** Focal somatic copy number alteration regions (SCNA) identified by GISTIC

A: Focal amplification (left) and deletion (right) regions

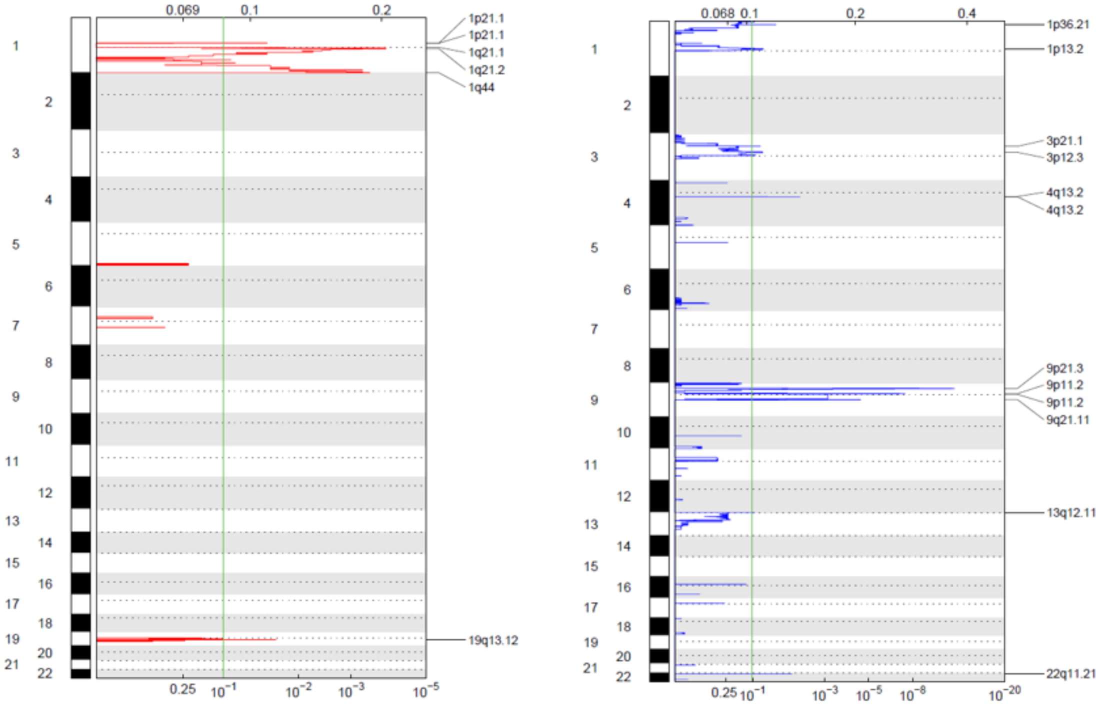

B: Focal SCNAs correlated with expression levels of key genes in the peak regions; *PBRM1* and *SETD2* expression in tumors without 3p21 deletion (n = 14) and in those with the deletion (n = 13). In B, data distributions are represented as boxplots where the line in the middle is the median, the first and the third quartiles are the box edges, the upper whisker extends from the edge to the largest value equal to 1.5 × IQR from the edge (where IQR is the inter-quartile range) and the lower whisker extends from the edge to the smallest value at most 1.5 × IQR of the edge, while data beyond these whiskers represent the outliers. The p-value of a two-sided Wilcoxon rank sum test is shown in the figure.

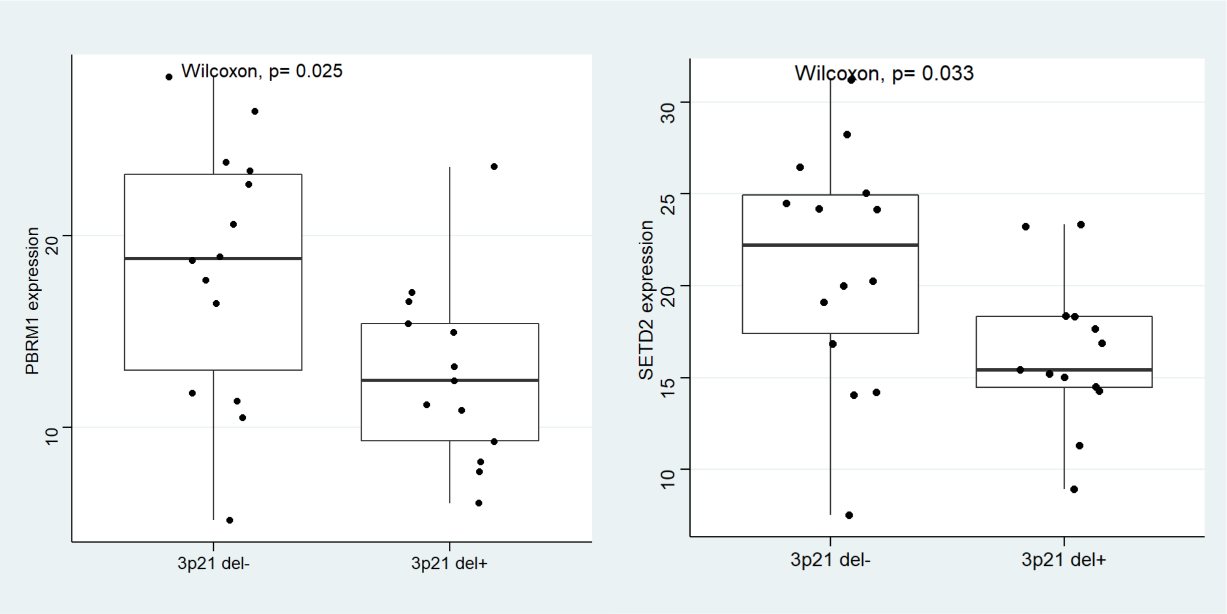

Supplementary Figure 7: Chromosomal somatic copy number alteration regions (SCNA) events in tumors without known drivers.

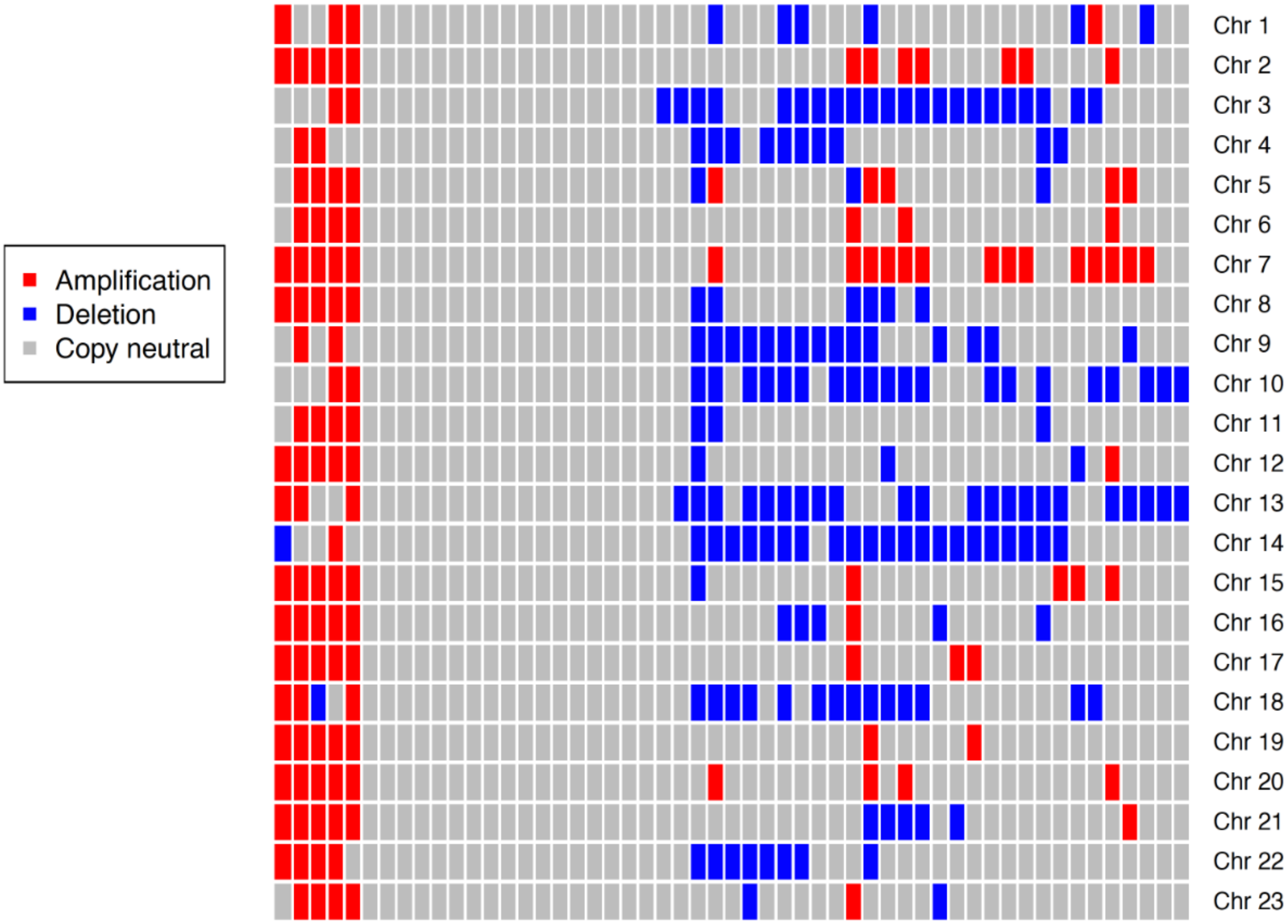

Supplementary Figure 8: Genomic events in relation to expression levels of the most relevant genes. In A–C, data distributions are represented as boxplots where the line in the middle is the median, the first and the third quartiles are the box edges, the upper whisker extends from the edge to the largest value equal to 1.5 × IQR from the edge (where IQR is the inter-quartile range) and the lower whisker extends from the edge to the smallest value at most 1.5 × IQR of the edge, while data beyond these whiskers represent the outliers. The p-value of a two-sided Wilcoxon rank sum test is shown in the figure.

A: *PBRM1* expression in tumors without *PBRM1* alteration (n = 24) and in those with the alteration (n = 3).

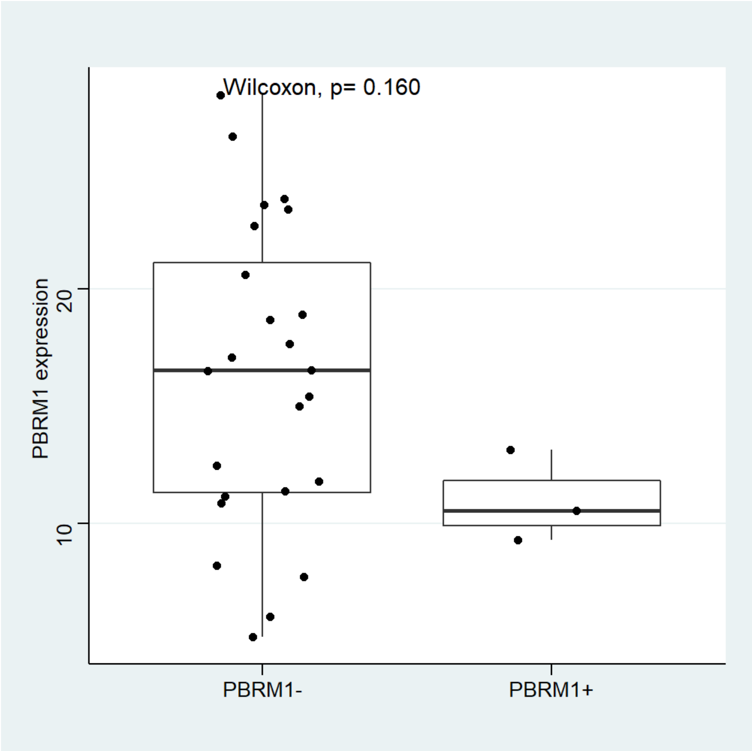

B: *CDKN2A* expression in tumors without *CDKN2A/2B* alteration (n = 23) and in those with the alteration (n = 4).

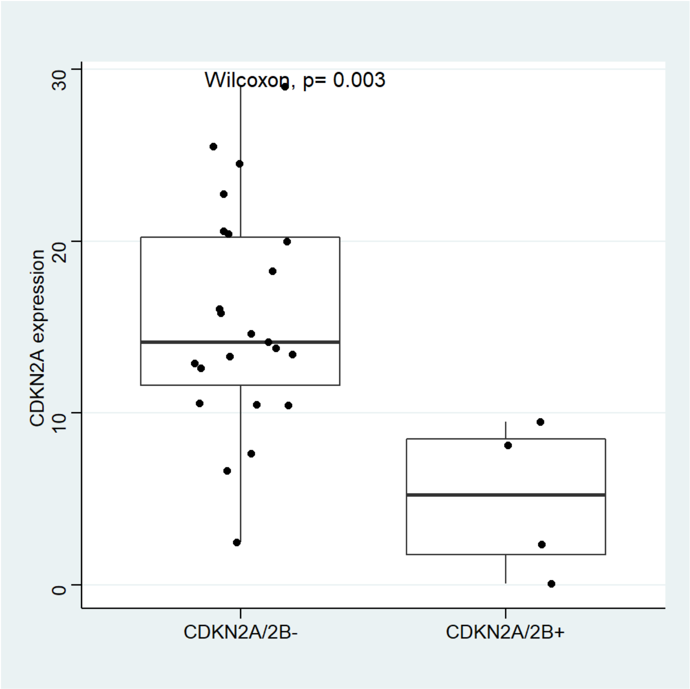

C. *SMARCB1* expression in tumors without arm-level chromosome 22q deletion (n = 18) and in those with the deletion (n = 9).

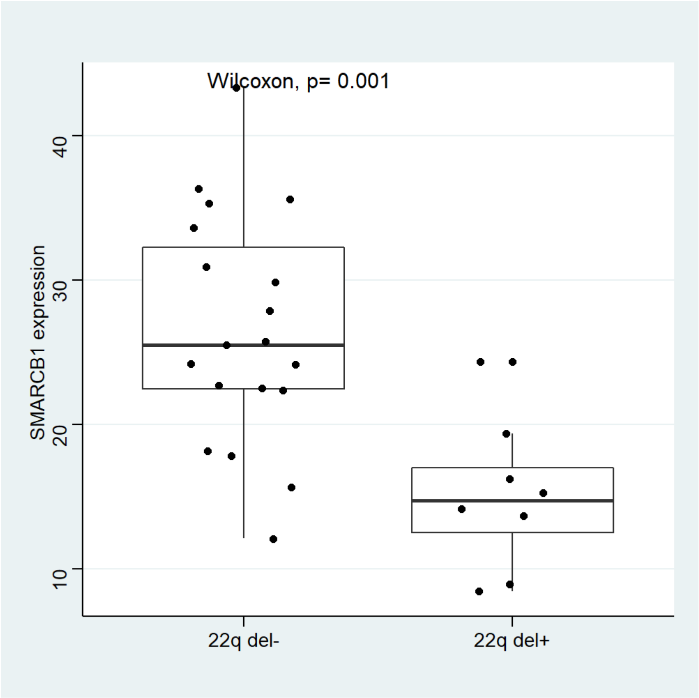

Supplementary Figure 9**:** The focal deletion of the *CDKN2A* region in the three paired tumor and metastasis samples. B: Blood (germline), no deletion detected; TM: thoracic metastasis, R: recurrence, LM: lymph node metastasis; *CDKN2A* region deletion was seen in all three paired tumor samples (TM, R, and LM).

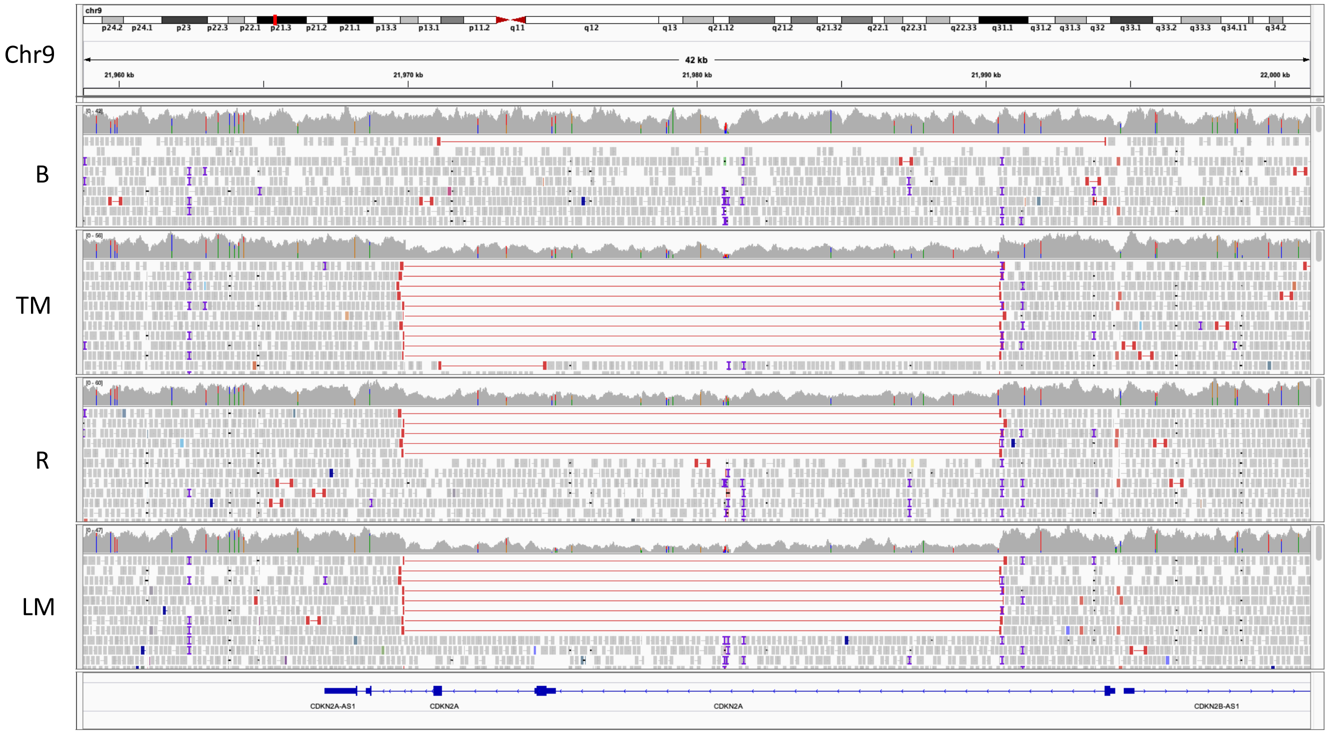

Supplementary Figure 10**:** Images (×200 magnification) of haematoxylin and eosin (H&E) and immunohistochemical (IHC) staining of the two patients with IDH1 mutations (a-c patient P59, d-f patient P26). a and d: H&E staining; b: High expression of BRACHYURY; c and f: Strong positive for CYTOKERATIN; e: Positive EMA staining.

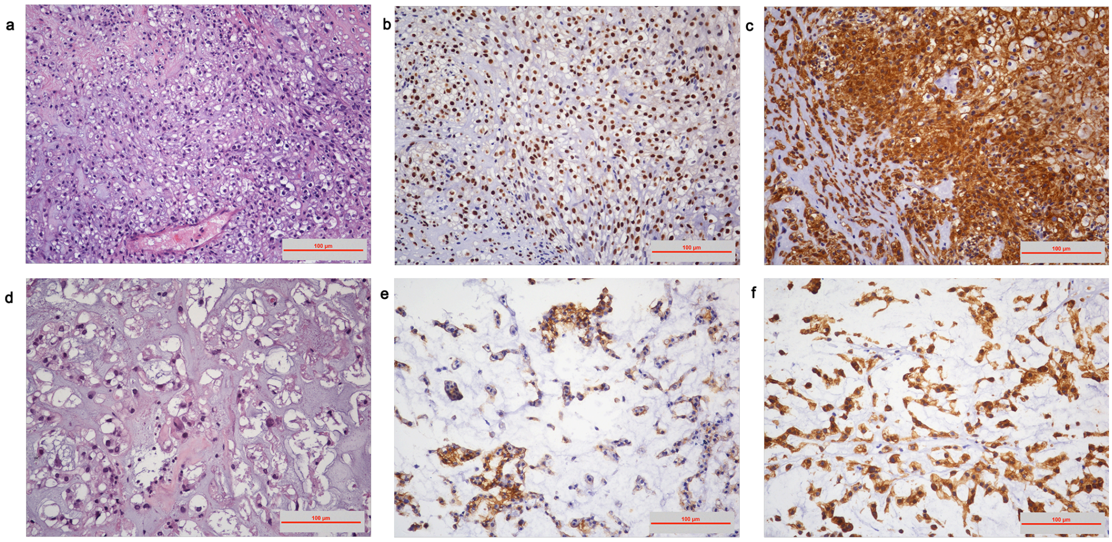

Supplement: Supplementary file 1 — Supplementary Information [file 41467_2021_21026_MOESM1_ESM.pdf]
