# Supplementary material for: Whole genome sequencing of skull-base chordoma reveals genomic alterations associated with recurrence and chordoma-specific survival
Source: Nat Commun. 2021 Feb 3;12:757. doi: 10.1038/s41467-021-21026-5 (PMC7859411; doi:10.1038/s41467-021-21026-5)
Supplement: Supplementary file 3 — Reporting Summary [file 41467_2021_21026_MOESM3_ESM.pdf]

nature research

Corresponding author(s): Yazhuo Zhang
Last updated by author(s): Nov 4, 2020

Reporting Summary

Nature Research wishes to improve the reproducibility of the work that we publish. This form provides structure for consistency and transparency in reporting. For further information on Nature Research policies, see our Editorial Policies and the Editorial Policy Checklist.

Statistics

For all statistical analyses, confirm that the following items are present in the figure legend, table legend, main text, or Methods section.

n/a	Confirmed	
☐	☒	The exact sample size (n) for each experimental group/condition, given as a discrete number and unit of measurement
☐	☒	A statement on whether measurements were taken from distinct samples or whether the same sample was measured repeatedly
☐	☒	The statistical test(s) used AND whether they are one- or two-sided<br>*Only common tests should be described solely by name; describe more complex techniques in the Methods section.*
☐	☒	A description of all covariates tested
☐	☒	A description of any assumptions or corrections, such as tests of normality and adjustment for multiple comparisons
☐	☒	A full description of the statistical parameters including central tendency (e.g. means) or other basic estimates (e.g. regression coefficient) AND variation (e.g. standard deviation) or associated estimates of uncertainty (e.g. confidence intervals)
☐	☒	For null hypothesis testing, the test statistic (e.g. F, t, r) with confidence intervals, effect sizes, degrees of freedom and P value noted<br>*Give P values as exact values whenever suitable.*
☒	☐	For Bayesian analysis, information on the choice of priors and Markov chain Monte Carlo settings
☐	☒	For hierarchical and complex designs, identification of the appropriate level for tests and full reporting of outcomes
☐	☒	Estimates of effect sizes (e.g. Cohen's d, Pearson's r), indicating how they were calculated

Our web collection on statistics for biologists contains articles on many of the points above.

Software and code

Policy information about availability of computer code

Data collection	Patient clinical and demographic data was collected using Microsoft Excel (V16), which was added in the manuscript.
Data analysis	All computer softwares used for bioinformatical and statistical analyses in this study were described in Methods. Maftools (v1.6.05), SigProfiler (v0.0.5.77), Meerkat (v0.189), dNdScv (HYPERLINK "https://github.com/im3sanger/dndscv/releases/tag/0.1.0" v0.1.0), pyclone (v0.13.1), facets (v0.5.6), ShatterSeek (v0.4), PennCNV-seq (v1.0.4), Canvas (v1.31), BWA (VN:0.7.8-r455), GATK Picard (v2.18), MoCCA-SV (V0.2), Svaba (v1.1.0), Breakdancer (v1.4.5), Delly (v0.8.1), Manta (v1.4.0), BAM-matcher (2016 version), sentieon (sentieon-genomics-201711.02), SomaticSeq (v2.7.2),<br>IGV (v2.3.61), GATK HaplotyperCaller, UnifiedGenotyper LeftAlignAndTrimVariants (v3.8).

For manuscripts utilizing custom algorithms or software that are central to the research but not yet described in published literature, software must be made available to editors and reviewers. We strongly encourage code deposition in a community repository (e.g. GitHub). See the Nature Research guidelines for submitting code & software for further information.

Data

Policy information about availability of data

All manuscripts must include a data availability statement. This statement should provide the following information, where applicable:

- Accession codes, unique identifiers, or web links for publicly available datasets
- A list of figures that have associated raw data
- A description of any restrictions on data availability

Whole genome sequencing data can be accessed through GSA (https://bigd.big.ac.cn/gsa/) under Accession# PRJCA002098). All other relevant data are available on request from the corresponding author （YZ). We have added the data availability statement in the manuscript with the accession number. We have also added the download site for TCGA data.

[illegible]

Dual use research of concern

Policy information about dual use research of concern

Hazards

Could the accidental, deliberate or reckless misuse of agents or technologies generated in the work, or the application of information presented in the manuscript, pose a threat to:

No	Yes	
☒	☐	Public health
☒	☐	National security
☒	☐	Crops and/or livestock
☒	☐	Ecosystems
☒	☐	Any other significant area

Experiments of concern

Does the work involve any of these experiments of concern:

No	Yes	
☒	☐	Demonstrate how to render a vaccine ineffective
☒	☐	Confer resistance to therapeutically useful antibiotics or antiviral agents
☒	☐	Enhance the virulence of a pathogen or render a nonpathogen virulent
☒	☐	Increase transmissibility of a pathogen
☒	☐	Alter the host range of a pathogen
☒	☐	Enable evasion of diagnostic/detection modalities
☒	☐	Enable the weaponization of a biological agent or toxin
☒	☐	Any other potentially harmful combination of experiments and agents
